# The Brain Structure and Intrinsic Characters of Falsification Thinking in Conditional Proposition Testing

**DOI:** 10.3389/fnhum.2021.684470

**Published:** 2021-08-23

**Authors:** Meng Zhang, Li Wang, Feng Zou, Yufeng Wang, Xin Wu

**Affiliations:** ^1^Department of Psychology, Xinxiang Medical University, Xinxiang, China; ^2^Department of Psychiatry, Henan Mental Hospital, The Second Affiliated Hospital of Xinxiang Medical University, Xinxiang, China

**Keywords:** Wason's selection task, voxel-based morphology, microstate, temporal lobe, falsification thinking

## Abstract

Wason's selection task (WST) as a representative of the field of conditional proposition testing has been explored by multiple disciplines for more than 50 years, but the neural basis of its key falsification thinking remains unclear. Considering that the accuracy of individuals in WST has stability over time, we believe that falsification thinking has a specific brain structural basis and intrinsic neural characteristics. To test this hypothesis, we studied individuals who were able to complete the WST using T1-weighted MRI (using voxel-based morphology (VBM) analysis) and resting electroencephalogram (EEG) (using microstate analysis, which can reflect stable cognitive characteristics of individuals) techniques. First, VBM analysis found that, compared with the verification group, the gray matter volume (GMV) of the left inferior temporal gyrus and the right superior temporal region of the falsification group was larger, whereas the GMV in the cerebellum of the verification group was significantly larger than that of the falsification group. Subsequently, the results of the microstate analysis of the resting EEG data showed that the contribution of class A of the falsification group, which is closely related to the language network, is significantly higher than that of the verification group. Our structural MRI and resting EEG results consistently show that the structure and intrinsic activity pattern of the temporal lobe in individuals with falsification thinking are specific. Furthermore, the findings may provide potential insights into the role of the temporal lobe (which is also a brain region of language processing) in thought.

## Introduction

Throughout history, humans have never stopped making hypotheses and evaluating evidence. Conditional propositions are common forms in which individuals make assumptions and are expressed in the if... then... form. For example, if he comes to my home, I will be very happy. Generally, the first clause of a statement (usually represented by the letter p) specifies a condition, and the second clause (represented by the letter q) specifies a result. Conditional propositions contain four forms: modus ponens (MP), denying the antecedent (DA), affirming the consequent (AC), and modus tollens (MT). Each form of conditional propositions testing is illustrated in Table 1.

**Table 1:** Four forms of conditional propositions testing.

**Name**	**Form**	**Name**	**Form**
MP	If p, then q	AC	If p, then q
	p		q
	Therefore q		Therefore, uncertainty
DA	If p, then q	MT	If p, then q
	Not p		Not q
	Therefore, uncertainty		Therefore, not p

As an early and classical paradigm for the test of conditional propositions, Wason's (1966) selection task (WST) has attracted a large number of interdisciplinary scholars to study it. Previous studies have found that only about 10% or less of the participants correctly selected the pair of MP and MT when completing the WST (Wason, 1977). The low accuracy of the WST was replicated across time and space (Evans et al., 1993; Oaksford, 1994; Stenning, 2004). Recently, a meta-analysis of WST also showed that the correct percentage of abstract WST was 19% (Ragni et al., 2017). In terms of WST itself, the correct answers MP and MT have the function of falsifying conditional propositions, which is also the core of the WST solution (Popper, 1959; Wason, 1968; Ragni et al., 2018). In the process of conditional proposition testing, the individual needs to evaluate the evidence under the condition statement framework of if... then..., which is the process of reasoning and decision. In fact, it is impossible to completely prove a conditional statement, but falsification is possible. For example, we cannot prove that birds can fly by saying that sparrows can fly, but we can deny that birds can fly by saying that ostriches cannot fly. In a conditional statement of absolute probability, a counterexample is sufficient to falsify the conditional proposition. Therefore, it is very important to understand the falsification thinking to complete the WST.

Although the accuracy rate of WST is too low, and there is a large stable systematic bias, some influential models have tried to explain the reasons. An earlier insight model/mental model (Johnson-Laird and Wason, 1970) believed that, in the task of WST, the individuals who selected the pairs of MP and AC were the most, and these individuals were those who did not have insight. In the process of checking the cards, they would only choose those that matched the rules, so the so-called matching bias or verification bias would occur (Wason, 1968; Evans, 1972, 1998). Moreover, those individuals who can correctly choose MP and MT have insight, and they will focus on checking whether the evidence has a falsifiable function in the process of the conditional proposition testing. In addition, there may also be individuals who have partial insight, who will examine all the evidence one by one, who will verify the conditional proposition if all the evidence supports it, and who will falsify the conditional proposition if they fail to do so. Obviously, the insight model emphasizes the importance of individual differences in solving the WST, that is, individuals with insights are capable of solving the WST, although the process of solving may have a difference in that whether falsified thinking plays a primary driving role in WST solving. Another influential model is the inference-guessing model (Klauer et al., 2007), which holds that the solution of WST depends on the probability that subjects understand if... then... statements as conditional rather than as biconditional, and the probability that subjects understand if... then... statements as forward inference. Different from the insight model that only individuals who are partially specific have the ability to solve the WST, the inference-guessing model theory puts more emphasis on the influence of logic and probability on the WST solution. Furthermore, there are also some theories or hypotheses for the interpretation of the WST, such as the thematic facilitation effect (Wason and Johnson-Laird, 1972; Evans and Johnson-Laird, 2003), the context effect (Almor and Sloman, 1996, 2000; Staller et al., 2000; Girotto et al., 2001), the necessity and sufficiency content (Thompson, 1995; Fairley et al., 1999; Hilton et al., 2005), the pragmatic reasoning schema theory (Cheng and Holyoak, 1985), the heuristic-analytic theory (Wason and Evans, 1975; Evans and Wason, 1976; Evans, 1984, 1989, 2006), and the social contract theory (Cosmides, 1985, 1989; Cosmides and Tooby, 1992, 2013; Fiddick et al., 2000); some of these theories or hypotheses support the insight model, while others support the inference-guessing model. To sum up, the insight model, which emphasizes individual differences, believes that it is difficult for individuals who are not logically trained to have falsification thinking, and individuals who can correctly complete WST may have specificity. While the inference-guessing model, which puts more emphasis on the situation/state, believes that the coding of conditional propositions and knowledge and experience of individuals will affect their evaluation process of evidence. Still, we need more multidisciplinary evidence to better understand the WST.

In recent years, cognitive neuroscience studies have provided evidence for the neural association of WST and conditional reasoning and have tested the relevant models to a certain extent. A task fMRI study on the conditional proposition testing directly explored the neural basis of individual falsification thinking and found that the activation level of the left middle frontal gyrus, the left inferior parietal lobule, the left precuneus, and the right cerebellum was closely related to the correct selection of cards of subjects with falsified functions (Liu et al., 2012). Reis et al. (2007) conducted fMRI studies using the social exchange content WST as the experimental material and they found high activation in the left frontal polar region and the left anterior temporal region. Another fMRI study found significant activation in the medial prefrontal cortex and the dorsolateral frontal–parietal cortex when participants completed the WST with social content (Canessa et al., 2005). Although the experimental materials in these studies are different, we can still find that the left frontal lobe may play an important role in solving WST. In addition, we also reviewed fMRI studies of conditional reasoning and found that the left frontal lobe, the left parietal lobe, and the left temporal lobe play important roles in the logical coding of conditional propositional, while activation of the right frontal cortex is more dependent on the content or context (Goel and Dolan, 2001, 2003, 2004; Knauff et al., 2003; Noveck et al., 2004; Canessa et al., 2005; Fangmeier et al., 2006; Prado and Noveck, 2007). Based on the results of the literature, we believe that the left cerebral cortex (including the frontal, parietal, and temporal lobes) plays a key role in the conditional proposition testing or the conditional reasoning problem solving. However, due to the limited literature, it is unclear what the response pattern of these brain regions is when individuals encode if... then... and whether these brain regions are closely related to falsified thinking.

Additionally, considering the low accuracy of WST and the stability of its behavioral results, combined with the fact that its behavioral results may be influenced by repeated stimuli (repeated resolution of WST), as well as the content and experience, based on previous studies, we boldly but reasonably believe that the logical naive individuals who can successfully resolve abstract WST may be specific at the brain level. Voxel-based morphological (VBM) analysis of the brain is a relatively mature method at present, which can characterize the structural features of the brain related to the stability behavioral characteristics (Christina et al., 2014). Furthermore, in the present study, a resting electroencephalogram (EEG) microstate technology emerging in recent years will also be used. Resting state EEG microstates originated from the phenomenological study of EEG. Researchers found that the resting EEG could be grouped into four microstate classes in a very stable manner (Lehmann and Skrandies, 1980; Lehmann, 1990; Koenig et al., 2002; Koenig, T. et al., 2005; Britz et al., 2010; Milz et al., 2016; Seitzman et al., 2016). Later, based on the behavioral and brain imaging studies, they gradually reveal the implications of the four EEG microstate classes: class A was related to the activity in the bilateral superior temporal gyrus and the middle temporal gyrus, which may reflect the verbal process; class B was associated with the activity of the bilateral lateral occipital lobe, which may reflect the cognitive process of visual representation; class C was related to the activity of the anterior cingulate cortex, insula, and inferior frontal gyrus, which may be closely related to the internal representation of the external information and the body state; and class D is associated with the activity of the right dorsolateral prefrontal, which was the key brain region of the dorsal attention network activity (Britz et al., 2010; Seitzman et al., 2016; Zanesco et al., 2019). The EEG microstate technology can reveal the spatial organizational characteristics and temporal dynamic changes of large-scale neural networks in the brain at the millisecond level, and it is considered as an excellent candidate for the study of the neural association of interindividual and intraindividual differences because it conforms to the stream of the consciousness theory of William James and the neuron working space model to a certain extent (James, 1890; Dehaene et al., 1998, 2003). Based on this, we believe that the use of the resting EEG microstate analysis is an effective technology to explore the brain functional networks with a higher temporal resolution, which can effectively supplement the results of structural MRI, and we can also expand the research field of resting EEG microstates to a certain extent. Here, we tried to use the aforementioned two methods to test the hypothesis that the logical naive individuals of abstract WST successful solution could be specific at the brain level, and tried to conduct a preliminary exploration of the insight model and the inference-guessing model.

## Methods

### Participants

Subjects for the structural MRI experiment: first, we tested 276 undergraduates aged 18–22 using the classic WST. According to the test results, we defined the subjects who chose MP and MT in the WST as the falsification group (FG, 46 subjects) and the subjects who chose MP and AC as the verification bias group (VG, 162 subjects). We removed all subjects who did not meet the requirements of MRI scans or who had systematically studied psychology or logic or who had previously participated in WST or conditional reasoning experiments, and called the remaining subjects one by one to ask if they would like to participate in subsequent MRI experiments. Finally, 25 subjects (16 women) in the FG and 57 (34 women) subjects in the VG completed all the experimental procedures, and their MRI data were included in the analysis. All subjects gave their written informed consent in accordance with the Declaration of Helsinki (1991). The ethics committee of Xinxiang Medical University approved the protocol for this study.

Subjects for the resting state EEG experiment: another 387 subjects aged 18–21 completed the classic WST first and were then divided into FG (47 subjects) and VG (178 subjects) groups according to the same criteria of MRI experiments. Any systematic psychology or logic or previous participation in WST or conditional reasoning experiments were excluded, and the remaining subjects were invited to participate in the EEG experiment on a voluntary basis. Finally, 26 subjects (14 women) in the FG and 90 subjects (67 women) in the VG completed all the experimental procedures, and their EEG data were included in the analysis. All subjects gave their written informed consent in accordance with the Declaration of Helsinki of 1975. The ethics committee of Xinxiang Medical University approved the protocol for this study.

### Behavioral Assessments

**Figure 1:**
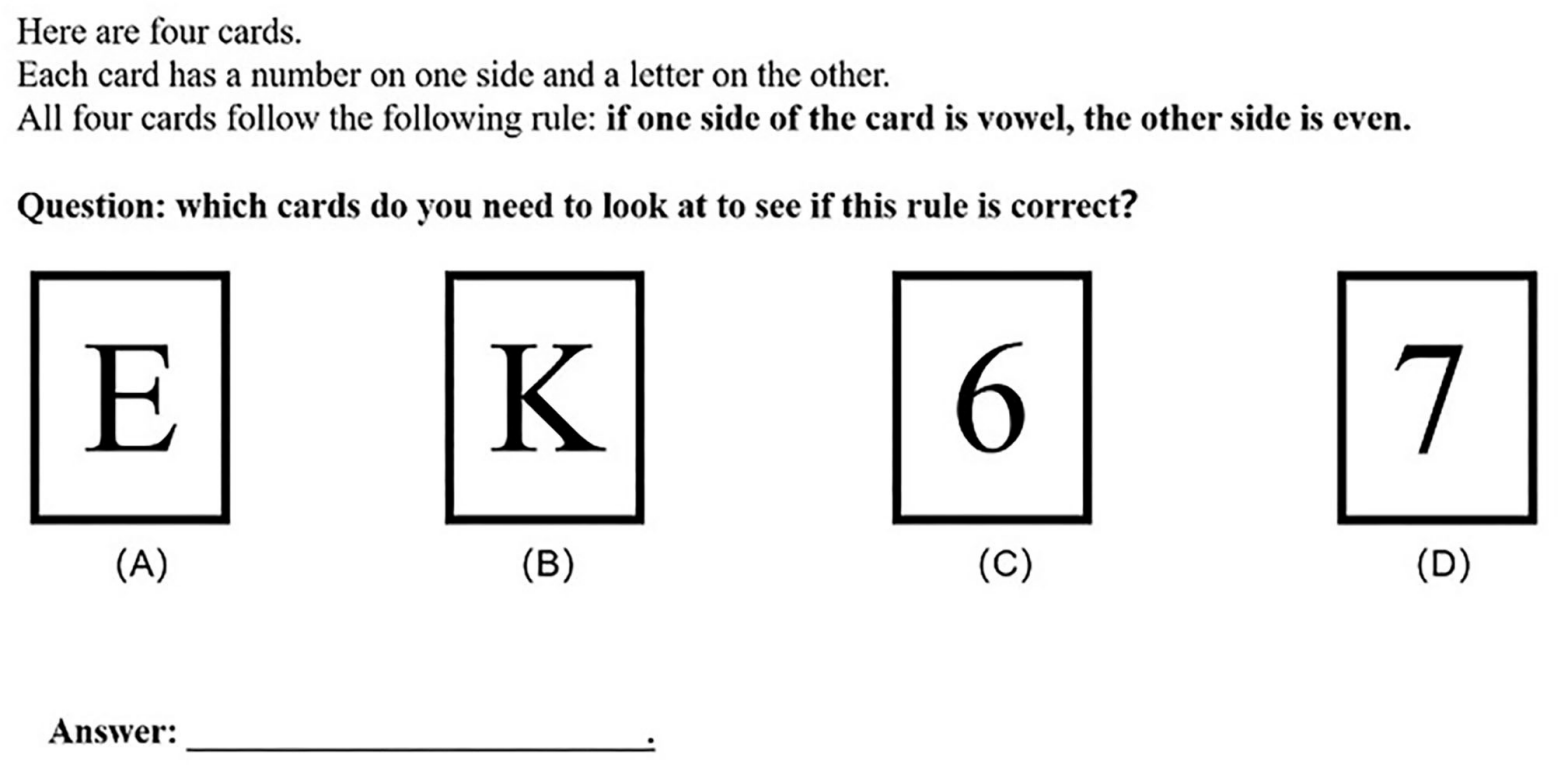
Illustration of the classical Wason's selection task in this study.

A classic WST was used as the experimental material for screening subjects. During the experiment, the subjects were shown four cards with a letter on one side and a number on the other, along with a conditional rule “if one side of the card is vowel, then the other side is even.” The task of subjects was to determine which cards they needed to look at in order to verify the conditional rule. The detailed information of the experimental material is shown in Figure 1.

Based on previous studies (Wason, 1966; Reich and Ruth, 1982; Liberman and Klar, 1996; Liu et al., 2012), and taking into account the diversity of the answers of participants in completing the WST, MP and MT pairing selection is the correct answer of the WST. In this task, the subjects had to flip over cards with a vowel and odd number to prove the logical falseness of the conditional rule. The subjects who can correctly complete WST are considered to have falsification thinking, which is defined as FG. In a conditional statement of absolute probability, a counterexample is sufficient to falsify the conditional proposition. Therefore, it is very important to understand the falsification thinking to complete the WST.

In addition, the matching selection of MP and AC is the answer with the highest proportion of subjects, and a common explanation for such a choice is that the subjects have a verification bias, so we define the subjects with answers of MP and AC as VG.

### Data Acquisition

#### T1 Data Acquisition

We used a 3.0T Siemens Trio scanner to collect the structural MRI data. Scan parameters are as follows: repetition time = 1,900 ms; inversion time = 900 ms; flip angle = 9; echo time = 2.52 ms; slices = 176; thickness = 1.0 mm; resolution matrix = 256 × 256; voxel size = 1 × 1 × 1 mm.

#### Resting State EEG Data Acquisition

Continuous EEG data for 5 min were obtained by 64-channel EEG data recording equipment (Neuroscan). The 64 Ag–AgCl scalp electrodes are arranged on the elastic cap according to the international 10–20 system. During the data collection process, the subjects were asked to open their eyes and look at the fixation point on the screen in front of them. The impedance of all electrodes was kept below 5 kΩ, and the sampling frequency was 500 HZ, and the band-pass filter was 0.1–100 Hz.

### Data Analysis

#### Structural MRI Data Analysis

The T1 images were processed by using SPM12 (Wellcome Department of Cognitive Neurology, London, UK) and implemented in MATLAB (MathWorks Inc., Natick, MA, USA). First, we used spm12 to render each T1 image to exclude the gross anatomical abnormalities. Then, the reorientation of the images was manually fixed to the anterior commissure. After that, we used the new segmentation feature in SPM12 to segment images of each subject into the following six tissue types: gray matter (GM), white matter, cerebrospinal fluid, the skull, the soft tissue outside the brain, and air and other things outside the head. Whereafter, the diffeomorphic anatomical registration through the exponentiated Lie (DARTEL) algebra in SPM12 was performed for registration, normalization, and modulation, and this procedure was conducted repeatedly until a best study-specific template was generated. Subsequently, the Jacobian determinants were used to modulate the image intensity of each voxel in order to make sure the conservation of regional differences in the absolute amounts of GM. Then, all registered images were transformed to the Montreal Neurological Institute (MNI) space. In the end, the normalized modulated GM images were smoothed with a 10-mm full-width at half-maximum Gaussian kernel to increase the signal-to-noise ratio.

After T1 data preprocessing, the SPM12 was used to perform the statistical analyses of the GMV data. In the whole-brain analyses, the two-sample *t*-test was subject to reveal the significant differences between the FG and VG in the brain structure morphology. For all analyses, the significant threshold was set at *p* < 0.05 [a combination threshold of voxel level at *p* < 0.01, and a cluster size of more than 812 voxels was obtained, which corresponded with a corrected *p* < 0.05 (using AlphaSim correction)].

#### Resting State EEG Data Analysis

EEGLAB (Delorme and Makeig, 2004) was used to preprocess the EEG data of all subjects. First, we checked the EEG data of each subject by visual examination to ensure that all electrode points were not in the neck or face, and we manually removed the portions that drifted larger. Then, we used the spherical alpine method to correct the bad channels. After that, the continuous EEG data were filtered by the band pass of 2 and 20 Hz. Subsequently, the independent component analysis (ICA) method was subjected to correct the artifacts including eye movements, blinks, electromyography, electrocardiography, and any non-physiological artifacts. The current dataset was remontaged against the average reference and segmented into 2,000-ms epochs, whose amplitude ranges from −80 to +80 μV. Then, the topographic atomizer and agglomerate hierarchical clustering algorithm [T-AAHC, (Brunet et al., 2011; Santarnecchi et al., 2017)] was used to compute the topographical representations of the EEG datasets of each subject in order to identify clusters with similar topographical configurations. In this step, the polarity of each map is ignored. Further, we use the Cartool (https://sites.google.com/site/cartoolcommunity/) to determine the optimal number of clusters at the individual level and group level. In the end, the microstate alternating sequence was re-expressed from each epoch, during that the temporal window smoothing method (half-size was 30 ms, the besag factor was 10, and the window frames <30 ms were rejected) was applied (Murray et al., 2008).

Before statistical analysis, we calculated and extracted the following indexes for each microstate class of each subject:

- The duration: This index represents the mean time coverage (in ms) of the same microstate class on the consecutive original maps, and in this study, we corrected the duration of each microstate class by using the following formula according to the previous study (Schlegel et al., 2012):

**Duration**_corrected_ **=** **Duration**_origin_^*^ **(occurrence** **+** **time coverage) / occurrence**

- The occurrence rate: This index was defined as the number of occurrences of a given microstate class per second.
- The contribution: The contribution of each microstate represents the percentage of the occupied total analysis time for the given microstate class.
- Transition probability: This index reflects the frequency intensity of a one-way or two-way switch between any two microstate classes.

Finally, the two-sample *t*-test was used to examine the differences between the FG and VG in the index of duration, occurrence rate, contribution, and transition probability. For all analyses, all *p*-values were corrected by the Bonferroni (*p* < 0.05^*^1/n).

## Results

### Behavioral Results

According to the test results, the subjects who chose MP and MT in the WST answered correctly, and we defined the subjects as the falsification group. In the structural MRI experiment, 46 subjects correctly completed the task, and the accuracy was 16%. In the resting state EEG experiment, 47 subjects correctly completed the task, and the accuracy was 12%. The low accuracy is consistent with the results of previous studies (Evans et al., 1993; Oaksford, 1994; Stenning, 2004; Ragni et al., 2017). Additionally, the individuals who selected the pairs of MP and AC were the most, and they would only choose those that matched the rules, hence the so-called matching bias or verification bias, which contains 162 subjects in the structural MRI experiment and 178 subjects in the resting state EEG experiment.

### Structural MRI Results

**Figure 2:**
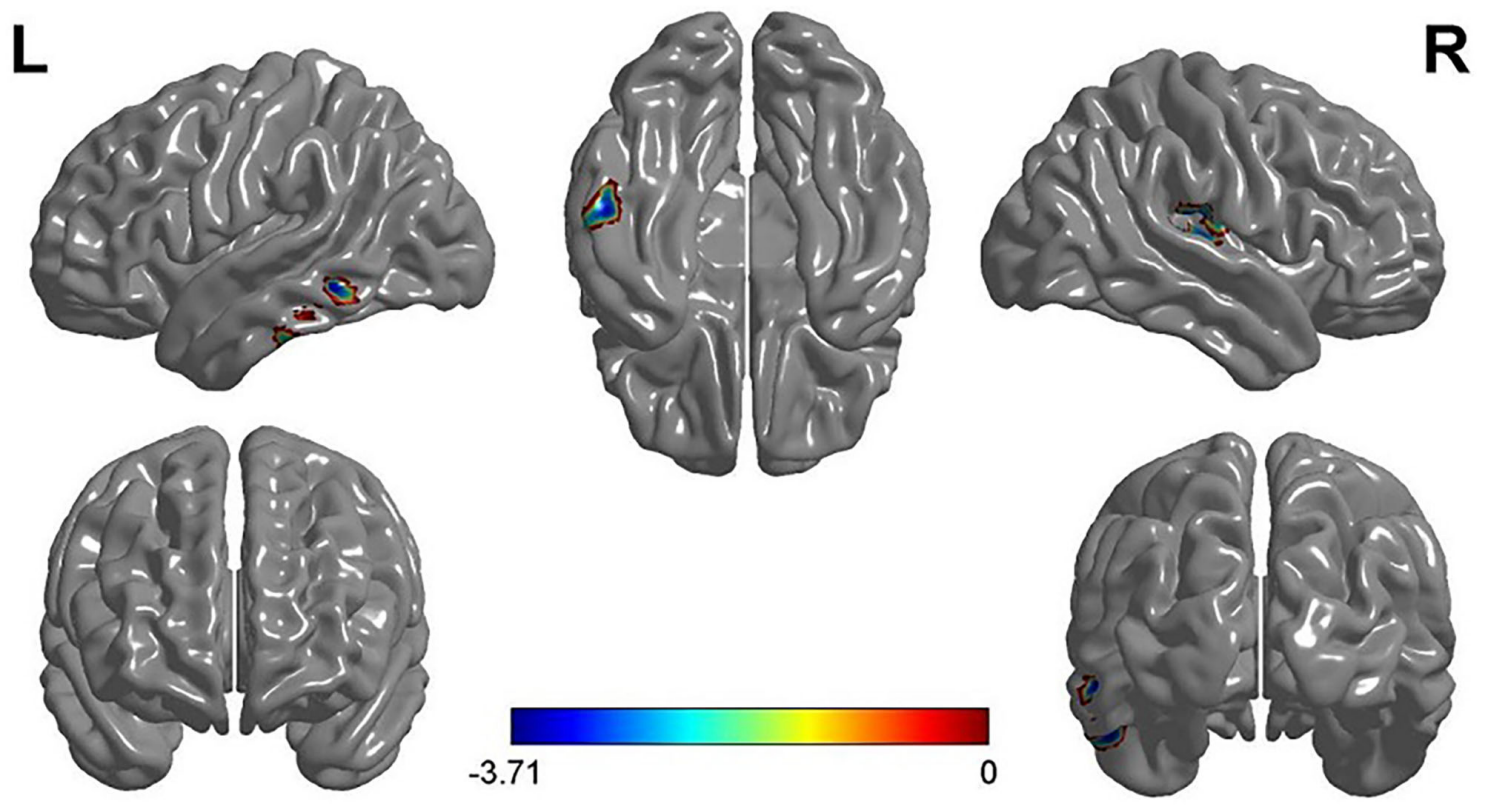
The results of VG vs. FG in the VBM analysis and the gray matter volume (GMV) of the left inferior temporal gyrus and the right superior temporal gyrus of VG were smaller than that of FG. Note: L, left; R, right.

The two-sample *t*-test analysis of the GMV of FG and VG showed that the GMV of FG in the left inferior temporal gyrus (MNI coordinates: −52.5, −27, −24) and the right superior temporal gyrus (MNI coordinates: 42, −19.5, 16.5) was significantly larger than that of VG. Furthermore, the GMV of VG in the right cerebellum (MNI coordinates: 6, −91.5, −3.5) was significantly larger than that of FG (see Figure 2 and Table 2).

**Table 2:** Brain regions showing significant difference in GMV by comparison of VG vs. GF.

**Brain region**	**MNI coordinates**			**Voxels size**	**Peak T value**
	**X**	**Y**	**Z**		
**FG > VG**					
Left inferior temporal gyruss	−52.5	−27	−24	835	3.71
Right superior temporal gyrus	42	−19.5	16.5	1176	3.08
**VG > FG**					
Right cerebelum_crus2	6	−91.5	−3.5	2094	3.6

### Resting State EEG Results

**Figure 3:**
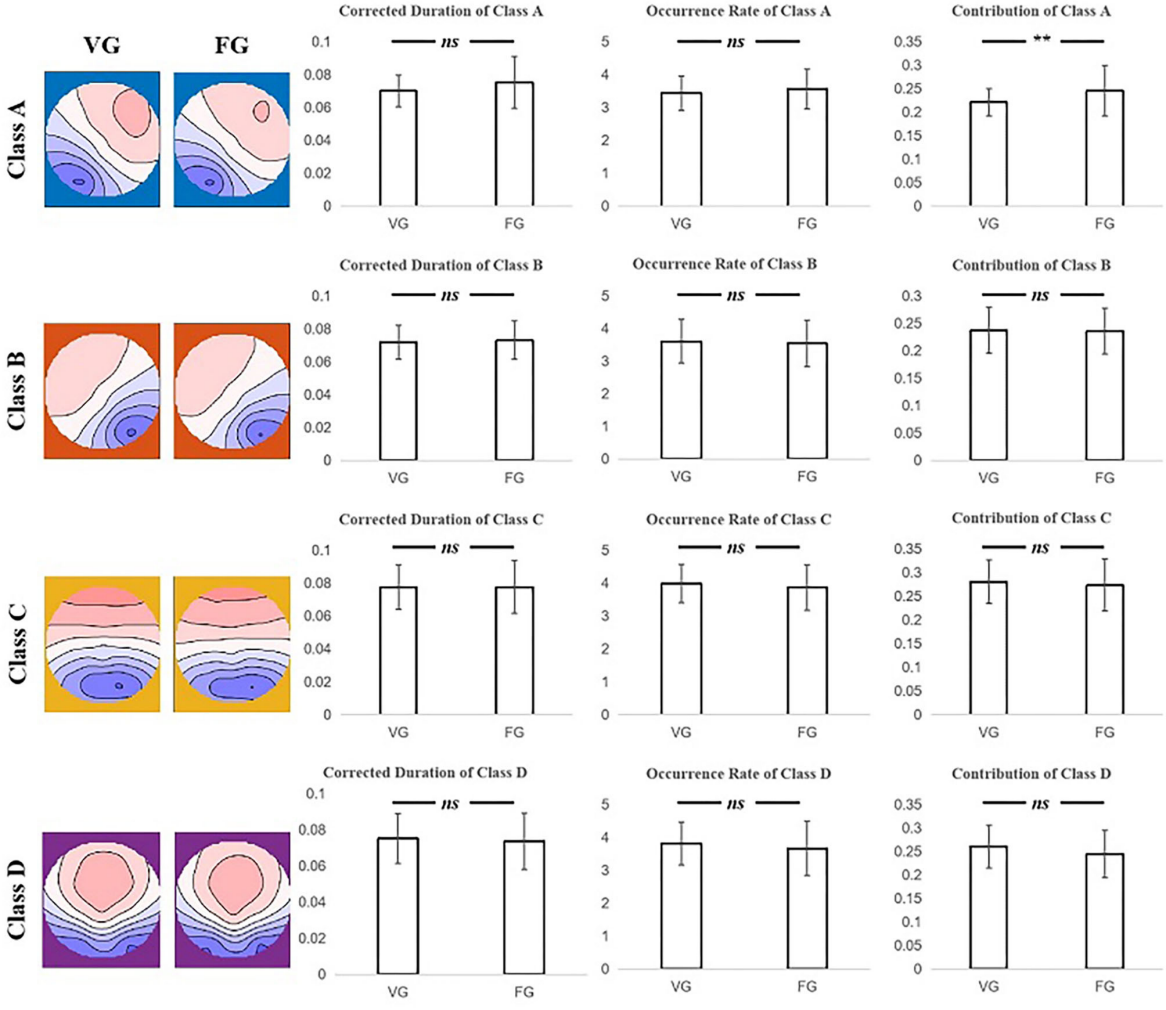
The results of the two-sample *t*-test for corrected duration, occurrence rate, and contribution index of each microstate class between the VG and FG. Only the contribution of class A of FG significantly larger than that of VG was found. The first column on the left illustrates that the microstate map of two groups of each class. Note: ns means no significant difference, ** means *p* < 0.005.

The two-sample *t*-test was subjected to examine the differences between the FG and VG in the index of duration, occurrence rate, contribution, and transition probability. Moreover, the results showed that only the contribution of class A of the VG was significantly less than that of FG (*t* = −3.079, *p* = 0.003) (see Figure 3 and Table 3).

**Table 3:** The means and standard deviation (SD) of transition probability between any two microstate classes.

	**VG**		**FG**	
	**Mean**	**SD**	**Mean**	**SD**
**From A to B**	0.07	0.01	0.07	0.01
**From A to C**	0.08	0.01	0.09	0.02
**From A to D**	0.07	0.01	0.08	0.02
**From B to A**	0.07	0.01	0.07	0.02
**From B to C**	0.08	0.02	0.08	0.02
**From B to D**	0.08	0.02	0.08	0.02
**From C to A**	0.08	0.01	0.09	0.02
**From C to B**	0.09	0.02	0.08	0.02
**From C to D**	0.10	0.02	0.09	0.02
**From D to A**	0.07	0.02	0.08	0.02
**From D to B**	0.08	0.02	0.08	0.02
**From D to C**	0.09	0.02	0.08	0.02

## Discussion

In this study, a large number of screened subjects were included to preliminarily explore the structural basis and intrinsic functional characteristics of the brain of falsification thinking. Analysis of VBM and resting state EEG data found that the bilateral temporal lobes of individuals with falsification thinking had larger GMV and larger contribution of class A related to speech network. We will then discuss the results and their implications.

Previous studies on the resting state EEG have found that microstate class A is associated with the activation of language processing-related brain regions such as the bilateral superior temporal gyrus and the bilateral middle temporal gyrus. In this study, we found that the contribution of class A with FG is significantly higher than that with VG, which is consistent with our VBM research results. The temporal lobe, which is closely related to speech processing, is also frequently discussed in reasoning (Coello et al., 2013; Gainotti, 2014). Parsons and Osherson (2001) conducted brain imaging studies using complex conditional propositions as experimental materials. They found the activation of the inferior frontal gyrus and the bilateral middle temporal speech areas in deductive reasoning and they speculated that these brain areas may constitute deductive modules. Goel et al. (1997, 1998, 2000), Goel and Dolan (2003) explored the neural basis of individual deductive reasoning by using event-related fMRI and found that large neural networks, including bilateral prefrontal lobe, left temporal lobe, and bilateral parietal lobe, may be the functional basis of logical reasoning. Acuna (2002) also found the activation of bilateral prefrontal lobes, auxiliary motor regions, insula, precuneus, and lateral posterior parietal cortex in individuals performing logical reasoning. Subsequently, Heckers et al. (2004) used a similar paradigm and reported the activation of the bilateral frontal-parietal temporal system. In addition, several studies of reasoning based on patients with brain injury have supplemented the evidence for reasoning related to the basis of brain function. Some studies have reported that lesions in the left temporal lobe and the whole brain have significant effects on logical reasoning (Caramazza, 1976; Golding, 1981; Read, 1981; Whitaker, 1991; Langdon and Warrington, 2000). In the above research context, the temporal lobe has been reported in a large number of studies on logical reasoning, and some literature has concluded that the temporal lobe is sensitive to processing abstract materials in logical reasoning (McCarthy and Warrington, 1990; Goel, 2005), which is also consistent with the double-processing theory (Sloman, 1996; Evans, 2003). However, it should be noted that the temporal lobe is the most frequently concerned and discussed brain region in theory of mind (ToM) reasoning (Fletcher et al., 1995; Goel et al., 1995). Therefore, we inferred that the temporal lobe is sensitive to more abstract or upper materials in logical reasoning processing, and it may play a role in monitoring and strategy adjustment (meta-reasoning) in logical reasoning. In addition, based on the conclusions of this study, we found that individuals capable of addressing WST are specific at both the level of brain structure and the level of intrinsic activity patterns, which to some extent supports the insight model.

In addition, our findings are consistent with the previous studies that more subjects chose pairs of MP and AC, while the right cerebellum GMV of this population (VG) was larger than that of FG. The resting state EEG did not find significant differences in the intrinsic brain activity characteristics between VG and FG. The cerebellum plays an important role in sensory and perception discrimination, working memory, mental imagery, decision-making, reasoning, monitoring, and cognitive flexibility (Kim et al., 1994; Mellet et al., 1995; Elliott and Dolan, 1998; Blakemore et al., 2001; Blackwood and Ffyche, 2004; Timmann and Daum, 2007). In recent years, some studies have found that the cerebellum plays an important role in implicit sequence learning and prediction, including verbal implicit sequence learning and prediction (Ackermann et al., 2007; Ito, 2008; Timmann et al., 2010; Jutta et al., 2018). According to the mental model theory (Johnson-Laird, 1983; Johnson-Laird et al., 2015), the reasoning process of individuals is not a rule-based process but an operational process of the mental representation of premises in spatial memory. Moreover, some studies believe that individuals will encode and integrate premises according to daily probabilities in reasoning and then make conclusions (Oaksford and Chater, 2001, 2007). Therefore, we believe that, consistent with the previous studies, the findings of the cerebellum in VG indicate that most of the individuals are deviating from rules when testing conditional propositions.

In conclusion, we used the classical abstract WST as the experimental material, selected individuals who chose the pair of MP and MT (FG) and individuals who chose the pair of MP and AC (VG) as subjects, and used structural MRI and resting state EEG to conduct data-driven exploration on the structural basis and intrinsic functional characteristics of the brain related to falsification thinking. The results showed that the structure and intrinsic function of the bilateral temporal lobe might be closely related to the falsified thinking. Individuals who can solve WST tasks, who are also individuals with falsification thinking, have a specific brain basis. This result partly explains why the low accuracy rate of WST is characterized by cross-time and partly supports the insight model (Johnson-Laird and Wason, 1970).

But going back to the WST itself, whether in work or in scientific research, the preference and evaluation of an individual for evidence under the conditional proposition framework are very important for the decision. As suggested by the results of this study, some individuals may be more sensitive to falsified evidence due to the specificity of their neural basis, but it is not ruled out that some individuals may examine the falsified function of evidence under certain circumstances. Therefore, on the basis of this preliminary study, we believe that the solution of the following problems may play an important role in a deeper understanding of WST and falsification thinking: (1) for logically naive individuals, whether they will look for counterexamples of propositions at the level of consciousness in the process of solving WST; (2) how individuals encode if... then..., and what are the cognitive neural mechanisms of the preference of individuals for the evaluation of evidence under the framework of conditional propositions; (3) whether the process of the conditional proposition test is affected by the confidence of subjects in the conditional proposition (such as the rationality of inductive reasoning, counterfactual probability, and other factors), thus influencing the judgment criteria of subjects for signals (cards or evidence).

## Data Availability Statement

The original contributions presented in the study are included in the article/supplementary material, further inquiries can be directed to the corresponding author.

## Ethics Statement

The studies involving human participants were reviewed and approved by The ethics committee of Xinxiang Medical University. The patients/participants provided their written informed consent to participate in this study.

## Author Contributions

MZ: conceptualization, methodology, formal analysis, investigation, data curation, writing (original draft), writing (reviewing and editing), and visualization. LW: methodology, formal analysis, investigation, data curation, and writing (reviewing and editing). FZ and YW: investigation and writing (reviewing and editing). XW: methodology, formal analysis, investigation, data curation, writing (reviewing and editing), and visualization. All authors contributed to the article and approved the submitted version.

## Conflict of Interest

The authors declare that the research was conducted in the absence of any commercial or financial relationships that could be construed as a potential conflict of interest.

## Publisher's Note

All claims expressed in this article are solely those of the authors and do not necessarily represent those of their affiliated organizations, or those of the publisher, the editors and the reviewers. Any product that may be evaluated in this article, or claim that may be made by its manufacturer, is not guaranteed or endorsed by the publisher.
